# P02.77. Case series to evaluate the efficacy of facial acupuncture to decrease skin roughness and depth of wrinkles in the glabellar area

**DOI:** 10.1186/1472-6882-12-S1-P133

**Published:** 2012-06-12

**Authors:** M Brodsky, S Prikhodko

**Affiliations:** 1 Stamford Hospital, Columbia University, Stamford, USA; 2 University of California Los Angeles, Los Angeles, USA

## Purpose

To evaluate the efficacy of facial acupuncture to decrease skin roughness and depth of wrinkles in the glabellar area.

## Methods

Nine subjects aged 40-60 were recruited to participate in the study. Each participant received facial acupuncture treatments 2 times a week for 4 weeks for a total of 8 treatments. An impression of the glabellar area was taken on 3 occasions: 1 month prior to treatment, on the first day of treatment prior to the procedure, and at the conclusion of the final treatment. In this manner each of the participants served as his or her own control. On each of the 3 occasions, a Cuderm replica-locating ring localized the glabellar area, using the root of the nose and eyebrow ridges as landmarks. A negative replica of the skin surface was made using Silfo, a dental replica material.

## Results

Surface roughness, number of wrinkles, and depth of wrinkles of the 3 serial glabellar impressions for each patient were analyzed by surface profilometry. There was no statistically significant change in any of the parameters measured. Qualitatively, 3 subjects noted a moderate improvement in wrinkles in the forehead area, 3 subjects noted a minimal improvement in wrinkles in the forehead area, and 3 subjects noted no improvement or worsening in wrinkles in the forehead area. Qualitative “eyeball” analysis of before and after glabellar impressions by an expert in surface profilometry noted a slight improvement in the replicas of 5 of the subjects and no change or slightly rougher appearance of the replicas of 4 of the subjects.

## Conclusion

This pilot study did not demonstrate statistically significant improvement in wrinkles with the facial acupuncture treatment protocol. Patient self-report and qualitative analysis of the impressions suggests that the treatment may benefit some individuals. A larger study may be warranted.

